# Breaking down the barriers to universal health coverage

**DOI:** 10.2471/BLT.17.190991

**Published:** 2017-02-01

**Authors:** Piyasakol Sakolsatayadorn, Margaret Chan

**Affiliations:** a Ministry of Public Health, Tivanond Road, Nonthaburi 11000, Thailand.; b World Health Organization, Geneva, Switzerland.

*The* *2030* *agenda for sustainable development* calls on the international community to prioritize the needs and rights of vulnerable populations, so that no one is left behind.^1^ The sustainable development goals (SDGs) are supremely ambitious, broad-based in their scope and strongly focused on the root causes of human misery, including the multiple interacting forces that make populations vulnerable to ill-health and premature death.

Vulnerability is often associated with poverty, but it is also shaped by political processes and policies, legislation that excludes population groups or criminalizes certain behaviours, and social attitudes that marginalize, stigmatize and discriminate. Vulnerable populations addressed in this issue include remote rural populations and the urban poor, children affected by drought and conflict, people living with the human immunodeficiency virus (HIV) and at risk of tuberculosis, persons with physical disabilities, undocumented migrant workers and gender minorities. The SDG target for universal health coverage requires that the health needs of these and other vulnerable groups be met. As universal health coverage entails social protection against financial hardship caused by health-care costs, it also contributes to the overarching SDG objective of poverty alleviation.

Papers in this issue cover a range of practical strategies for reaching vulnerable populations and addressing the multiple social, economic and environmental determinants of health. Research in Ethiopia shows how examining the effects of drought and conflict on the prevalence of wasting in children can guide the design of population-wide interventions.^2^ Gavi, the Vaccine Alliance, has developed a tool for monitoring equitable vaccine coverage, using equity benchmarks that reflect the ambitions of the sustainable development agenda.^3^ In Thailand, a programme for modifying the homes of people with disabilities proved technically and financially feasible, with support from government subsidies.^4^ In Nepal, using peers to contact people living with HIV for tuberculosis screening resulted in a high participation rate and the identification of a considerable number of HIV-positive tuberculosis patients, illustrating one way to break through the barriers of discrimination.^5^

Models for extending service coverage stress the importance of education, training and community engagement. Enhanced recruitment, training, supervision, and compensation of community health workers rapidly improved coverage with maternal and child health services in rural areas of Liberia.^6^ Brazil has used a package of incentives to recruit physicians to work in remote and deprived areas and to improve the primary health-care infrastructure, leading to better working conditions and better quality of care.^7^

Political commitment can be decisive. A paper on the fate of underserved and marginalized populations during donor transition shows how limited political commitment can lead to the persecution of vulnerable groups, pointing to the need to engage key populations in planning, implementing, and monitoring the transition.^8^ A paper on the health of the urban poor shows how governments often play a role in aggravating the predicament of this large and vulnerable group.^9^ In Thailand, the growing cost of subsidizing migrant workers’ health care, through exemption of user fees on a humanitarian basis, prompted the government to initiate an innovative health insurance scheme for migrants.^10^

Transgender people are another vulnerable group facing social and legal barriers that are pervasive in both health-care settings and broader society. WHO and its partners have developed a range of tailored guidance for health practitioners and policy-makers to better protect the health and rights of transgender people.^11^ Finally, more needs to be done to meet the long-term rehabilitation needs of people who suffer disabilities during disasters.^12^

Taken together, these papers illustrate the place that health occupies in *The 2030 agenda for sustainable development*. Health is an end-point that reflects the success of multiple other goals. Because the social, economic and environmental determinants of health are so broad, progress in improving health is a reliable indicator of progress in implementing the overall agenda. In the final analysis, the ultimate objective of all development activities – whether aimed at improving food and water supplies or making the urban poor safe – is to sustain human lives in good health. Moreover, all health targets can be reliably measured using established scientific methods. As shown by the papers in this issue, disease burdens and their causes can be measured, the impact of specific interventions can be assessed, and changes over time can be tracked.

The inclusion of an SDG target for reaching universal health coverage, including financial risk protection, affirms the power of health to build fair, stable, and cohesive societies while also contributing to poverty alleviation. The target provides a unifying platform for moving towards all other health targets through the delivery of integrated, people-centred services that span the life course, bring prevention to the fore and protect against financial hardship. Universal health coverage is the ultimate expression of fairness and one of the most powerful social equalizers among all policy options.
